# Emergence of Carbapenemase Genes in Gram-Negative Bacteria Isolated from the Wastewater Treatment Plant in A Coruña, Spain

**DOI:** 10.3390/antibiotics13020194

**Published:** 2024-02-17

**Authors:** Mohammed Nasser-Ali, Pablo Aja-Macaya, Kelly Conde-Pérez, Noelia Trigo-Tasende, Soraya Rumbo-Feal, Ana Fernández-González, Germán Bou, Margarita Poza, Juan A. Vallejo

**Affiliations:** 1 Microbiology Research Group, Institute of Biomedical Research (INIBIC)-University Hospital of A Coruña (CHUAC)-Interdisciplinary Center for Chemistry and Biology (CICA)-University of A Coruña (UDC)-CIBER de Enfermedades Infecciosas (CIBERINFEC, ISCIII). Servicio de Microbiología, 3° planta, Edificio Sur, Hospital Universitario, As Xubias, 15006 A Coruna, Spain; mohammednasserma87@gmail.com (M.N.-A.); paabloaja@gmail.com (P.A.-M.); kelly.conde.perez@sergas.es (K.C.-P.); noeliatrigo@gmail.com (N.T.-T.); soraya.rumbo.feal@sergas.es (S.R.-F.); ana.fernandez.gonzalez@sergas.es (A.F.-G.); german.bou.arevalo@sergas.es (G.B.); 2 Microbiome and Health Group, Faculty of Sciences, Campus da Zapateira, 15071 A Coruna, Spain

**Keywords:** antimicrobial resistant bacteria, carbapenemase, Gram-negative bacteria, wastewater-based epidemiology, whole-genome sequencing, *bla*_KPC_

## Abstract

Wastewater treatment plants (WWTPs) are recognized as important niches of antibiotic-resistant bacteria that can be easily spread to the environment. In this study, we collected wastewater samples from the WWTP of A Coruña (NW Spain) from April 2020 to February 2022 to evaluate the presence of Gram-negative bacteria harboring carbapenemase genes. Bacteria isolated from wastewater were classified and their antimicrobial profiles were determined. In total, 252 Gram-negative bacteria carrying various carbapenemase genes were described. Whole-genome sequencing was conducted on 55 selected carbapenemase producing isolates using Oxford Nanopore technology. This study revealed the presence of a significant population of bacteria carrying carbapenemase genes in WWTP, which constitutes a public health problem due to their risk of dissemination to the environment. This emphasizes the usefulness of WWTP monitoring for combating antibiotic resistance. Data revealed the presence of different types of sequences harboring carbapenemase genes, such as *bla*_KPC-2_, *bla*_GES-5_, *bla*_GES-6_, *bla*_IMP-11_, *bla*_IMP-28_, *bla*_OXA-24_, *bla*_OXA-48_, *bla*_OXA-58_, *bla*_OXA-217_, and *bla*_VIM-2_. Importantly, the presence of the *bla*_KPC-2_ gene in wastewater, several months before any clinical case was detected in University Hospital of A Coruña, suggests that wastewater-based epidemiology can be used as an early warning system for the surveillance of antibiotic-resistant bacteria.

## 1. Introduction

Antibiotics are one of the most significant medical achievements of the 20th century. They can kill or inhibit the growth of bacteria that cause infectious diseases. The excessive and irresponsible use of antibiotics has led to bacterial pathogens developing antibiotic resistance (AR) and spreading, posing a threat to human health. It has been estimated that at least 700,000 deaths occur annually worldwide due to antibiotic-resistant bacteria (ARB). This is expected to reach 10 million deaths due to bacterial resistance by 2050, which could entail an estimated cost of USD 100 billion [1]. Therefore, the World Health Organization (WHO) and the USA Centers for Diseases Control and Prevention (CDC) have considered that AR is a global public health problem, especially carbapenem resistance in Gram-negative bacteria. Additionally, special attention should be paid to the ESKAPE bacteria group, including *Enterococcus faecium*, *Staphylococcus aureus*, *Klebsiella pneumoniae*, *Acinetobacter baumannii*, *Pseudomonas aeruginosa*, and *Enterobacter* spp. [2], especially if they harbor carbapenem resistance genes and Extended-Spectrum Beta-Lactamases (ESBL) genes, which are often responsible for hospital infections [3,4]. However, carbapenems (meropenem, imipenem, doripenem, and ertapenem), among the beta-lactam class of antibiotics, are used as last resort antibiotics for treating the most severe infections caused by multidrug-resistant bacteria [5,6,7]. Despite this, excessive carbapenem usage promotes a variety of resistance mechanisms that can hydrolyze carbapenems [8]. In Spain, carbapenem antibiotics rank as the fourth most prescribed group [9]. Carbapenem resistance can be mediated by four major mechanisms. These include the over-expression of genes encoding efflux pumps, the decrease in cell wall permeability, mutations on the antibiotic target, or the production of carbapenemases. The production of carbapenemases predominates worldwide as the main mechanism of resistance to carbapenems, which represents a significant health risk. These enzymes can break down carbapenems and other beta-lactam antibiotics and are mainly encoded on plasmids, which can be transmitted horizontally to other bacteria [5,6,7,10]. Carbapenemases belong to three Ambler classes: A, B, and D [5,6,10]. Class A carbapenemases include Guiana extended spectrum enzyme (GES), *Serratia marcescens* enzyme (SME), imipenem-hydrolyzing beta lactamase (IMI), *Serratia fonticola* carbapenemase-1 (SFC-1), and *K. pneumoniae* carbapenemase (KPC), all of which are clinically relevant. Class B carbapenemases include New Delhi metallo-beta-lactamase 1 (NDM), imipenem-resistant *pseudomonas* (IMP), Verona integron-encoded metallo-beta-lactamase (VIM), German imipenemase (GIM), and Seoul imipenemase (SIM). Class D carbapenemases include oxacillinase enzymes (OXAs), particularly the clinically relevant OXA-48 and its derivatives [5,6,10]. However, among carbapenemase enzymes, KPC, VIM, IMP, NDM, and OXA-48 are the most effective based on their carbapenem hydrolysis capacity and on their high ability to geographically spread [11]. Despite variations in the regional distribution of prevalence rates, these five carbapenemase genes are often identified in *Enterobacterales* [12]. 

Wastewater-based epidemiology (WBE) is an epidemiological approach that can be used for public health surveillance and to predict disease outbreaks at an early stage. WBE is based on the wastewater analysis of chemical or biological markers, such as pathogens, resistant genes, pharmaceuticals, and other health indicators, which can be pooled in one wastewater treatment plant connected to the local community [13]. Accordingly, the CDC [14] has considered WBE to be a key procedure for ARB surveillance. Therefore, wastewater surveillance systems have been developed in many places for tracking the transmission of different pathogens in populations, including resistant pathogens. This will provide insight into how humans, other animals, and the environment are exposed to this threat [14]. Wastewater treatment plants (WWTPs) are considered environmental hotspots for the spread of AR genes and AR bacteria [15]. WWTPs receive sewage from different sources, such as hospitals, households, and animal husbandries, which use antibiotics, biocides, disinfectants, metal residues, and pharmaceutical products, as well as different microorganisms. Antibiotics and other substances may induce selection pressure, promoting the horizontal gene transfer of resistance genes and leading to the dissemination of AR in wastewater [15,16]. The significance of wastewater as a reservoir for ARGs has been increasing over recent years, with regular reports on novel ARGs [17,18,19,20,21,22,23]. Several contemporary wastewater treatment plants are not able to remove these microorganisms completely, so the corresponding wastewater effluents or the sludges generated are potential sources of ARGs. Therefore, understanding the presence of clinically relevant ARB and their genes in wastewater could help in reducing their environmental circulation, thus protecting human and other animals’ health [15]. 

Previous studies have described the presence of diverse ARB and ARGs in wastewater, including carbapenem resistance [17,24,25,26,27,28,29,30]. However, due to the high significance of carbapenem-resistant bacteria, further studies on WWTPs are required to better understand molecular epidemiology. In the present study, we isolated and characterized a collection of carbapenem-resistant bacteria from wastewater samples obtained from the WWTP of A Coruña (A Coruña, Northwest of Spain) during the period April 2020–February 2022. This WWTP serves a population of ca. 400,000 inhabitants from the metropolitan area of A Coruña. Our findings revealed the presence of carbapenem-resistant bacteria in the wastewater. Interestingly, a collection of *K. pneumoniae* strains harboring *bla*_KPC_ genes was identified prior to clinical cases, which indicates the potential of wastewater-based epidemiology as an early warning system for community and hospital infections.

## 2. Results

### 2.1. Identification of Bacterial Isolates

**Figure 1:**
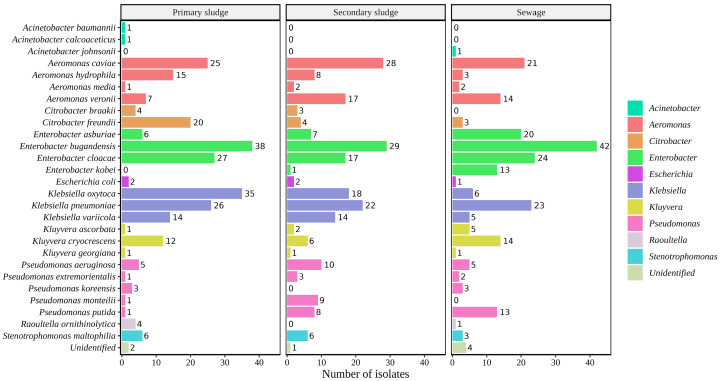
Representation of the species identified from the wastewater samples obtained from the WWTP of A Coruña. The bacterial isolates were isolated from three distinct sources within the WWTP: primary sludge, secondary sludge, and sewage. The numbers within the figure correspond to the count of bacterial isolates per individual species, while the colors employed in the figure represent the different genera.

In total, 706 bacterial isolates were identified. Among these, 32.43% (*n* = 229) were isolated from sewage, 36.7% (*n* = 259) from primary sludge, and 30.9% (*n* = 218) from secondary sludge in the WWTP. The identified species and their respective percentages are shown in Figure 1 and Table S1. 

### 2.2. Antimicrobial Susceptibility Testing

**Figure 2:**
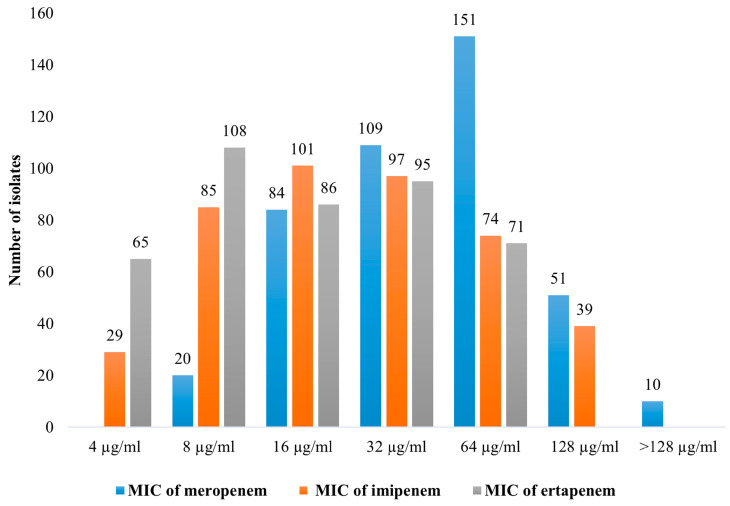
Number of carbapenem-resistant isolates found in this study and the corresponding MIC values for meropenem, imipenem, and ertapenem.

Among the 706 bacterial isolates, 425 were chosen for antimicrobial susceptibility testing according to their clinical relevance. The MIC results for these 425 bacteria are shown in Figure 2 and Table S1. In summary, MIC results for meropenem showed that all these 425 isolates were resistant (breakpoint for *Enterobacterales* R ≥ 4, *P. aeruginosa* and *Acinetobacter* spp. R ≥ 8, non-*Enterobacterales* R ≥ 16). MIC results for imipenem showed that 422 isolates were resistant (breakpoint for Enterobacterales R ≥ 4 µg/mL, *P. aeruginosa* and *Acinetobacter* spp. R ≥ 8 µg/mL, non-*Enterobacteriales* R ≥ 16 µg/mL). For ertapenem, only *Enterobacterales* were provided in accordance with CLSI standards. In our study, 360 *Enterobacterales* isolates (84.7% of the total) were described. All of them were resistant to ertapenem (breakpoint, R ≥ 2).

### 2.3. Carbapenemases Multiplex PCR Detection

**Figure 3:**
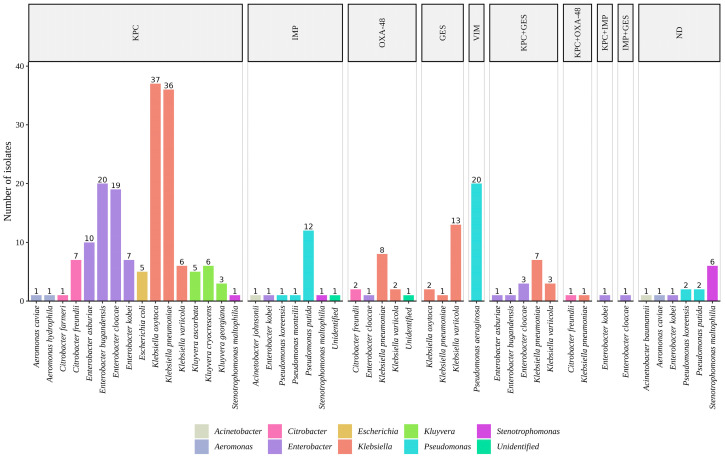
Distribution of carbapenemase genes detected in strains isolated from WWTP samples. The numbers within the figure indicate the count of bacterial species harboring carbapenemase genes. Each color corresponds to a genus. The ‘ND’ (not detected) abbreviation is used to indicate bacterial isolates lacking carbapenemase genes.

Carbapenemase genes were detected by PCR in 252 strains (95%) of the 265 isolates selected after antimicrobial susceptibility testing. Among the isolates, carbapenemase genes were detected in the following proportions: *bla*_KPC_ in 62.3% (165/265), *bla*_VIM_ in 7.5% (20/265), *bla*_IMP_ in 6.8% (18/265), *bla*_OXA-48_ in 5.6% (14/265), *bla*_GES_ in 6% (16/265), *bla*_KPC_ and *bla*_GES_ in 5.7% (15/265), *bla*_KPC_ and *bla*_OXA-48_ in 0.8% (2/265), *bla*_KPC_ and *bla*_IMP_ in 0.4% (1/265), *bla*_IMP_ and *bla*_GES_ in 0.4% (1/265). None of the bacteria tested positive for the carbapenemase gene *bla*_NDM_ (Figure 3).

Carbapenemase genes were not detected (ND) in 4.9% (13/265) of the isolates. A comprehensive list of all identified bacterial species and their corresponding carbapenemase genes is shown in Table S2.

### 2.4. Whole-Genome Sequencing

**Figure 4:**
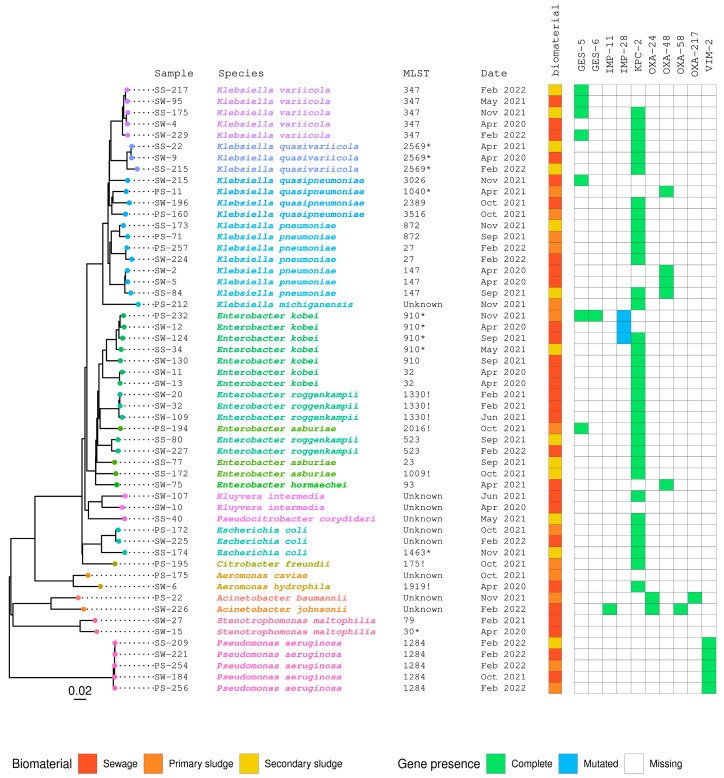
Phylogenetic tree representing the relationships among 54 isolates of Gram-negative bacteria and their respective STs and carbapenemase genes. The biomaterial column shows the sample origin from the WWTP of A Coruña, which include primary sludge (PS; brown), secondary sludge (SS; yellow), and sewage (SW; red). Asterisks (*) represent an allele with less than 100% identity, while the exclamation mark (!) indicates the nearest ST. The term “unknown” means that there is no information about the ST in the MLST database. The date column displays the isolation dates for each strain during the study period. The green squares represent the presence of a carbapenemase gene without mutations (100% identity), while the blue squares represent the presence of a carbapenemase gene with mutations (less than 100% identity). The white squares indicate the absence of carbapenemase genes.

Whole-genome sequencing was performed on 55 bacterial isolates chosen based on several factors, including clinical relevance, sampling site and date, MIC, and PCR results. To understand the genomic characteristics of these isolates, an analysis was conducted; the findings are summarized in Figure 4. Additional details can be found in Table S3. 

Twenty-one isolates of *Klebsiella* spp. were selected for sequencing. These were identified as *K. pneumoniae* (*n* = 7), *K. variicola* (*n* = 5), *K. quasipneumoniae* (*n* = 4), *K. quasivariicola* (*n* = 3), and *K. michiganensis* (*n* = 2). The seven *K. pneumoniae* strains belonged to three different sequence types (STs): ST147 (*n* = 3), ST27 (*n* = 2), and ST872 (*n* = 2).

The harbored carbapenemase genes presented 100% identity with those described. Two isolates of *Klebsiella pneumonia* (SW-2 and SW-5), which belonged to the ST147 group, carried the *bla*_OXA-48_ gene. Meanwhile, *K. pneumoniae* isolate SS-84, belonging to ST147, carried two types of carbapenemase genes, *bla*_KPC-2_ and *bla*_OXA-48_. Moreover, two *K. pneumoniae* isolates (PS-257 and SW-224) belonging to ST27 were found to have carbapenemase genes *bla*_KPC-2_. Two other isolates of *K. pneumoniae* (SS-173 and PS-71) belonged to ST872 carried the *bla*_KPC-2_ gene. Four *K. quasipneumoniae* isolates belonged to four different STs: ST3516 (PS-160), ST2389 (SW-196), ST1040* (PS-11), and ST3026 (SW-215). Carbapenemase genes harbored presents 100% of identity with those described. The PS-160 and SW-196 isolates were found to carry the *bla*_KPC-2_ gene. This isolate (PS-11) was found to carry the *bla*_OXA-48_ gene. Finally, the fourth isolate (SW-215) had the *bla*_GES-5_ gene. Five *K. variicola* isolates belonging to ST347 harbored carbapenemase genes with 100% identity. Two isolates of *K. variicola* (SS-175 and SW-229) carried two different carbapenemase genes. The SS-175 isolate was found to carry *bla*_GES-5_ and *bla*_KPC-2_ genes and the SW-229 isolate of *K. variicola* harbored the *bla*_GES-5_ and *bla*_KPC-2_ genes. In addition, SW-4, SW-95, and SS-217 isolates carried a single carbapenemase gene, *bla*_KPC-2_ in SW-4 and *bla*_GES-5_ in SW-95 and SS-217. The three *K. quasivariicola* isolates, SS-215, SW-9, and SS-22, showed less than 100% identity matches to the alleles of ST2569*. All of them carried the *bla*_KPC-2_ gene with 100% identity. The two *K. michiganensis* isolates (SS-168 and PS-212) were found to be unknown sequence types (STs) for the Multilocus Sequence Typing (MLST) database. Both isolates were found to carry the *bla*_KPC-2_ gene with 100% identity.

In addition, sixteen *Enterobacter* isolates were selected for sequencing. The isolates were classified as *Enterobacter kobei* (*n* = 7), *E. roggenkampii* (*n* = 5), *E. asburiae* (*n* = 3), and *E. hormaechei* (*n* = 1). Seven isolates of *E. kobei* were identified, and they were classified into different sequence types (STs). Among these isolates, four belonged to ST910*, one belonged to ST910, and two belonged to ST32. Notably, the ST910* isolates did not match the alleles of ST910* completely. These isolates, namely, PS-232, SW-12, SW-124, and SS-34, had less than 100% identity with the alleles of ST910*, whereas the ST910 isolate (SW-130) and the ST32 isolates (SW-11 and SW-13) showed a 100% identical match in the MLST database. The identities for carbapenemase genes in *E. kobei* isolates were determined as follows: *bla*_GES-6_ and *bla*_KPC-2_ exhibited 100% identity, whereas the *bla*_IMP-28_ gene showed slightly lower identity (99.59%) based on the CARD database. Among the *E. kobei* isolates, three were identified to harbor two carbapenemase genes each: PS-232, SW-124, and SW-130. PS-232 was found to carry both the *bla*_IMP-28_ and the *bla*_GES-6_ genes, SW-124 carried the *bla*_IMP-28_ and *bla*_KPC-2_ genes, and SW-130 was found to possess two copies of the *bla*_KPC-2_ gene. Four *E. kobei* isolates were identified, each carrying one carbapenemase gene. Specifically, SW-12 was found to contain the *bla*_IMP-28_ gene, SS-34 carried the *bla*_KPC-2_ gene, and both SW-11 and SW-13 isolates were observed to harbor the *bla*_KPC-2_ gene. Five isolates of *E. roggenkampii* were classified into different sequence types (STs). Within this set, three isolates (SW-20, SW-32, and SW-109) were recognized as the closest to ST1330! (! represents the closest to ST1330), while the two remaining isolates (SS-80 and SW-227) were assigned to ST523. All these isolates were identified as carriers of the *bla*_KPC-2_ gene, demonstrating a 100% match in the CARD database. Three isolates of *E. asburiae* were differentiated into three distinct sequence types (STs). Among these, the PS-194 isolate and SS-172 isolate were close to ST2016! and ST1009!, respectively, while the SS-77 isolate was classified under ST23. All the isolates exhibited carbapenemase genes with a complete match of 100% identity. Notably, the PS-194 isolate contained two carbapenemase genes, namely, the *bla*_KPC-2_ gene and the *bla*_GES-5_ gene, whereas both the SS-77 and SS-172 isolates carried a singular carbapenemase gene, specifically the *bla*_KPC-2_ gene. The *E. hormaechei* SW-75 isolate was identified as belonging to ST93. It was confirmed to carry the *bla*_OXA-48_ gene, showing complete identity at 100%.

Three *E. coli* isolates, namely, SW-225, PS-172, and SS-174, all carried the *bla*_KPC-2_ gene with complete identity at 100%. Interestingly, SW-225 and PS-172 isolates were not assigned to any known sequence types (STs) in the MLST database. In contrast, the SS-174 isolate was categorized to ST1463*, although its alleles had less than 100% identity. 

Moreover, two *Acinetobacter* isolates, SW-226 recognized as *A. johnsonii* and PS-22 identified as *A. baumannii*, were both characterized by unclassified sequence types (STs) in the MLST database. However, the SW-226 isolate harbored three carbapenemase genes: *bla*_IMP-11_, *bla*_OXA-58_ and *bla*_OXA-24_, while the PS-22 isolate harbored two carbapenemase genes: *bla*_OXA-217_ and *bla*_OXA-24_. All these carbapenemase genes exhibited complete identity at 100%.

In addition, one *Citrobacter freundii* (PS-195) isolate closely matched ST175! and carried the *bla*_KPC-2_ gene, showing a full 100% match. Additionally, the identified SS-40 isolate of *Pseudocitrobacter corydidari* did not correspond to any known ST in the MLST database, although it carried the *bla*_KPC-2_ gene, exhibiting a complete identity of 100%. Two *Kluyvera intermedia* isolates, namely, SW-107 and SW-10, were unknown STs in the MLST database. Notably, the SW-107 isolate carried two *bla*_KPC-2_ genes, both with 100% identity, whereas the SW-10 isolate lacked any carbapenemase genes. Two isolates of *Aeromonas*, namely, SW-6 (*A. hydrophila*) and PS-175 (*A. caviae*), were selected for sequencing. The SW-6 isolate was found to be closely related to ST1919!; conversely, the PS-175 isolate had an unknown ST in the MLST database. Notably, the SW-6 isolate carried the *bla*_KPC-2_ gene with a 100% identity match, whereas PS-175 did not harbor any carbapenemase genes. Two *Stenotrophomonas maltophilia* isolates were chosen for sequencing. The first isolate, SW-15, was assigned to ST30*, although its alleles did not exhibit a 100% identity match. Conversely, the second isolate, SW-27, belonged to ST79. Notably, neither isolate exhibited the presence of carbapenemase genes. Finally, five *Pseudomonas aeruginosa* isolates (SS-209, SW-221, SW-184, PS-256, and PS-254) were categorized as belonging to ST1284. All isolates exhibited the presence of the *bla*_VIM-2_ gene, showing complete identity at 100%.

### 2.5. Presence of Carbapenemase Genes in Patients from CHUAC

**Figure 5:**
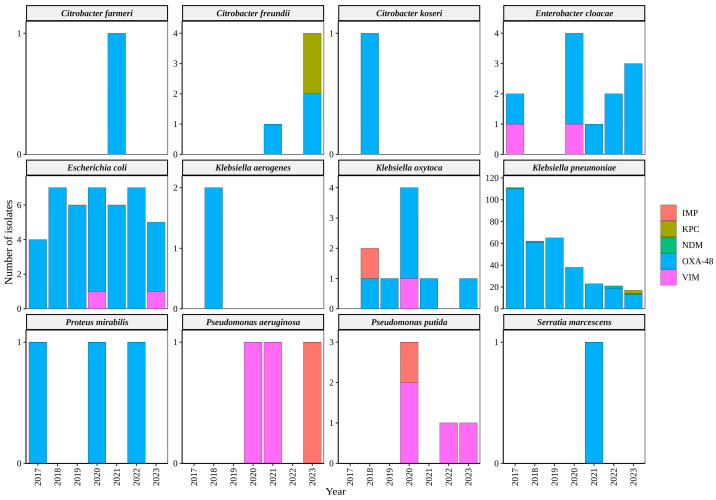
Timeline of bacterial clinical species carrying carbapenemase genes detected from 5 January 2017 to 11 September 2023 in patients from CHUAC (NW Spain).

In this study, we analyzed a dataset comprising 421 clinical bacterial isolates obtained from CHUAC, NW Spain. The isolates were collected over a span of approximately six years and eight months, from 5 January 2017 to 11 September 2023. Carbapenemase genes were identified in the clinical isolates, with the *bla*_OXA-48_ gene being the most prevalent, detected in 95% of isolates. This gene was consistently found from January 2017 to September 2023. Other carbapenemase genes were also detected in various years, including *bla*_VIM_ (2.6%, *n* = 11), *bla*_KPC_ (1.2%, *n* = 5), *bla*_NDM_ (1.2%, *n* = 5), and *bla*_IMP_ (0.71%, *n* = 3). The *bla*_KPC_ carbapenemase gene was identified for the first time among clinical isolates at our hospital (CHUAC) in June–July 2023, with occurrences found in three *K. pneumoniae* isolates and two *C. freundii* isolates. For specific, detailed information on the respective years, carbapenemase gene types, and the bacterial species that carried these genes, please refer to Figure 5 and Table S4.

## 3. Discussion

This research showed the presence of various carbapenemase genes (*bla*_KPC_, *bla*_VIM_, *bla*_IMP_, *bla*_OXA-24_, *bla*_OXA-48_, and *bla*_GES_) in Gram-negative bacteria that were isolated from the WWTP of A Coruña. This WWTP processes water from the metropolitan area of A Coruña, in the northwest of Spain. To the best of our knowledge, this investigation marks the first examination of ARGs at this specific site. The Gram-negative bacterial isolates were obtained from different sources within the wastewater treatment plant, including sewage, primary sludge, and secondary sludge. Our results indicated the presence and proliferation of these bacteria and their resistance genes in the WWTP. Similarly, previous studies have isolated a variety of bacteria and their resistance genes from all stages of WWTPs [29,31]. 

The Gram-negative bacterial isolates displayed significant diversity, with 70% of the isolates representing highly clinically relevant bacteria that are known to cause infections due to carbapenem resistance [2,3]. Among these clinically relevant carbapenem-resistant bacteria, *Enterobacteriaceae* such as *Klebsiella* spp., *Enterobacter* spp., *Citrobacter* spp., and *E. coli* accounted for 474 (95.8%) isolates, while *P. aeruginosa* and *A. baumannii* were represented by 20 (4%) and 1 (0.2%) isolate (s), respectively. By conducting PCR tests on the 265 selected isolates, we determined that 252 of them were positive for carbapenemase genes. Among these bacteria, the *bla*_KPC_ carbapenemase gene was the most prevalent carbapenemase type identified in this study, and the blaOXA-48 gene exhibited a lower prevalence. KPC-type carbapenemase are widely distributed and considered the most dominant carbapenemases among *Enterobacteriaceae* globally [32]. Nevertheless, several studies have reported a higher prevalence of *bla*_OXA-48_ among clinical isolates in multiple provinces of Spain [12,33,34,35]. However, despite extensive investigations, no evidence of the *bla*_KPC_ gene was detected among patients in the A Coruña metropolitan area (population of ca. 400,000) until June 2023. At this date, patients infected with bacteria harboring the *bla*_KPC_ gene were identified for the first time at the University Hospital of A Coruña. These clinical bacterial isolates carrying the *bla*_KPC_ gene included three *K. pneumoniae* isolates and two *C. freundii* isolates. (Figure 5 and Table S4). This fact indicates, once more, that wastewater-based epidemiology is an excellent tool for outbreak predictions, serving as an early warning system of bacterial resistance. Additionally, our investigation revealed the presence of *bla*_GES_ in bacterial isolates from WWTP, although this gene had not previously been detected within our hospital setting. Analysis of our hospital’s collected data suggests a probable emergence of this gene among future patients, similar to the pattern observed with *bla*_KPC_. The significant presence of the *bla*_KPC_ and *bla*_GES_ genes in our WWTP isolates indicates the potential presence of bacteria carrying these genes within the local community [33,36]. Consequently, bacteria isolated from WWTPs can serve as valuable indicators for predicting the existence of resistant bacteria in the local population [33,36]. In our study, the absence of the NDM in comparison to hospital isolates suggests a potential connection to various factors. These may encompass different selection pressures, dynamics of gene transfer (specifically, horizontal gene transfer), and the diverse nature of the bacterial population [37].

In a previous investigation, *bla*_KPC2_ was initially identified in a WWTP in Japan, despite its absence in clinical isolates. They proposed that the surveillance of AMR through WWTP could aid identifying the continuous dissemination of AMR in the environment and potentially serve as an early indicator of its spread in clinical settings and communities [38,39]. In another study conducted in Jeddah city, Saudi Arabia, researchers identified the presence of the carbapenemase gene *bla*_NDM-1_ in an *E. coli* strain (ST101) in wastewater for the first time. This gene was not detected in clinical isolates from the same area. The researchers proposed that the influx of international pilgrims to Jeddah could potentially have contributed to the spread of the *bla*_NDM-1_ gene in the wastewater treatment plant [40]. 

In our study, identification efforts revealed a variety of carbapenemase genes among the *Klebsiella* genus within the *K. pneumoniae* species complex (KpSC). These species include *K. pneumoniae* (deemed critically significant in clinical contexts), as well as *K. variicola*, *K. quasipneumoniae*, and *K. quasivariicola*, all linked to clinical infections [41,42]. In Spain, *K. pneumoniae* stands out as the predominant species harboring a significant number of carbapenemase genes, with *bla*_OXA-48_ being the most prevalent [43,44,45]. We detected *K. pneumoniae* isolates of sequence type ST147 carrying the *bla*_KPC-2_ gene, the *bla*_OXA-48_ gene, or both. The ST147 isolate has gained global recognition as a widely disseminated antimicrobial resistant clone, known to be associated with various carbapenemases (KPC, OXA-48, VIM, and NDM), and has been implicated in multiple nosocomial outbreaks worldwide [46]. Additionally, we detected *K. pneumoniae* strains of ST27 and ST872 carrying the *bla*_KPC-2_ gene, both STs known for their clinical significance [42,47]. We found *K. variicola* isolates of sequence type ST347 carrying either the *bla*_KPC-2_ gene, the *bla*_GES-5_ gene, or both. This specific ST347 strain, recognized for its clinical significance, has been observed to possess ESBL genes [48]. *K. quasipneumoniae* isolates, specifically ST3516 and ST2389, were identified as carriers of the *bla*_KPC-2_ gene. Additionally, within this species, ST1040 was found to harbor the *bla*_OXA-48_ gene, while ST3026 demonstrated carriage of the *bla*_GES-5_ gene. Furthermore, multiple *K. quasivariicola* isolates that belonged to ST2569 were identified as carriers of the *bla*_KPC-2_ gene. None of the *K. quasipneumoniae* STs or *K. quasivariicola* STs had been previously documented.

Additional types of carbapenemase genes have been detected in our work within the *E. cloacae* complex (ECC), such as *E. hormaechei*, *E. asburiae*, *E. roggenkampii*, *E. kobei*. ECC is frequently found in healthcare settings as a pathogen capable of causing a wide range of infections, such as pneumonia, septicemia, and urinary tract infections. Moreover, they are recognized for their multidrug resistance (MDR) [49,50,51]. Among them, we identified an *E. hormaechei* isolate of sequence type 93, carrying the *bla*_-OXA-48_ gene. This specific ST93 strain, found in a patient with a bloodstream infection, demonstrated the presence of carbapenemase genes including *bla*_NDM-1_ and *bla*_KPC-2_, in addition to several other resistance genes [52]. Isolates of *E. kobei* were identified as sequence type ST910 and displayed a spectrum of carbapenemase genes. One isolate notably presented a combination of *bla*_GES-5_, *bla*_GES-6_, and *bla*_IMP-28_ genes. Additionally, a separate isolate exclusively carried the *bla*_IMP-28_ gene, while another isolate demonstrated the presence of two carbapenemase genes, namely, *bla*_IMP-28_ and *bla*_KPC-2_. Lastly, two isolates were characterized by the sole presence of the *bla*_KPC-2_ gene. The ST910 was previously isolated from hospital wastewater, showing the presence of the *bla*_GES-24_ gene [53]. Moreover, its identification was documented in German surface waters, confirming the existence of the carbapenemase gene *bla*_VIM-1_ [54]. An additional *E. kobei* isolate, classified as ST32, exhibited the presence of the carbapenemase gene *bla*_KPC-2_. This strain, previously recovered from a clinical specimen in Colombia, also harbored the carbapenemase gene *bla*_KPC-2_ [55]. Isolates of *E. roggenkampii* were categorized into two distinct sequence types, namely, ST1330 and ST523, with both types exhibiting the presence of the *bla*_KPC-2_ gene. Notably, the ST1330 strain represents a novel detection, thus far unreported, whereas the ST523 strain, previously identified in a human clinical sample, harbored the *bla*_VIM-4_ carbapenemase gene [56]. *E. asburiae* isolates were classified into three distinct sequence types: ST2016, ST1009, and ST23. The ST2016 strain demonstrated the presence of both *bla*_GES-5_ and *bla*_KPC-2_, whereas the ST1009 strain carried the *bla*_KPC-2_ gene. Both STs indicate a novel detection. The ST23 strain was found to contain the *bla*_KPC-2_ gene, while prior clinical reports have indicated it association with the *bla*_VIM-4_ gene [56].

Isolates of *P. aeruginosa* were determined to be sequence type ST1284 and exhibited the presence of the carbapenemase gene *bla*_VIM-2_. Notably, this specific ST1284 strain was previously recovered from a patient diagnosed with ventilator-associated pneumonia in Brazil, and it was found to harbor the carbapenemase gene *bla*_VIM-7_ [57]. The *E. coli* strain identified in this study was classified as sequence type ST1463 and was found to harbor the *bla*_KPC-2_ gene. Notably, this particular ST1463 strain had previously been documented in China, originating from a patient specimen, and similarly exhibited the presence of the *bla*_KPC-2_ gene [58]. The *C. freundii* strain, classified as ST175, and *A. hydrophila* strain, categorized as ST1919, both carried the *bla*_KPC-2_ gene, representing a novel detection as these particular STs had not previously been observed. 

It is important to note that our sample collection from a single WWTP in our area may not fully represent the spread of carbapenemase genes in our country. Further studies are required to collect samples from different WWTP locations in Spain.

## 4. Materials and Methods

### 4.1. Sampling Site

The WWTP of A Coruña [59], located in the city of A Coruña (NW of Spain), collects sewage from the A Coruña metropolitan area. This metropolitan area includes A Coruña, Culleredo, Cambre, Oleiros, and Arteixo municipalities. The WWTP of A Coruña serves about 400,000 inhabitants, which constitutes ca. 0.78% of the total population of Spain. On average, the municipal sewage volume per day at the WWTP of A Coruña is about 135,000 m^3^.

### 4.2. Sampling, Isolation, and Bacterial Identification

The samples were collected from three locations within the WWTP of A Coruña, including untreated sewage (influent), primary sludge, and secondary sludge, during the period April 2020–February 2022. Each 500 mL sample was transported to the microbiology laboratory in a cooling box. Samples were collected using automatic sampling, as previously described [60].

Initially, the samples were diluted serially (1:10, 1:100, and 1:1000) in sterile water. Subsequently, 100 µL of each diluted sample was applied to MacConkey agar plates (BD Difco, Sparks, MD, USA) supplemented with 4, 8, and 16 µg/mL of meropenem (Sigma-Aldrich, Darmstadt, Germany), as well as mSuperCARBA TM plates (CHROMagar, Paris, France) [61]. All plates were incubated at 37 °C for 18–24 h. To represent the bacterial genetic diversity in the sample, a random collection of colonies was selected from these plates. These colonies were cultured on Luria Bertani (LB) agar medium plates, supplemented with 4 µg/mL of meropenem, and incubated at 37 °C for 18–24 h. Subsequently, each isolated species was identified using matrix-assisted laser desorption/ionization time-of-flight mass spectrometry (MALDI-TOF MS) (Bruker-Daltonik, Billerica, MA, USA). The identified isolates were stored in a solution of 20% glycerol and 80% LB broth at −80 °C for further analysis.

### 4.3. Antimicrobial Susceptibility Testing

The antimicrobial susceptibility testing was conducted using the broth microdilution test, involving meropenem (Sigma-Aldrich, Darmstadt, Germany), ertapenem (Sigma-Aldrich, Darmstadt, Germany), and imipenem (Sigma-Aldrich, Darmstadt, Germany) antibiotics, in accordance with the guidelines established by the Clinical and Laboratory Standards Institute (CLSI) [62]. Briefly, 100 µL of cation-adjusted Mueller–Hinton (M-H) broth 2 (Merck Millipore, Darmstadt, Germany) was added to the 96-well polystyrene microtiter sterile plate. Serially diluted concentrations of meropenem, imipenem, or ertapenem ranging from 0.25 to 128 µg/mL were established. In each plate, wells without antibiotics were included as positive controls of growth, and wells without inocula and antibiotics were used as negative control. The bacterial suspension at 0.5 McFarland (1 × 10^8^ CFU/mL) was diluted 1:20 to achieve 5 × 10^6^ CFU/mL, and 5 µL was added to the plate wells as an inoculum. The plates were placed in a plastic bag and incubated at 37 °C for 18 h. Minimum inhibitory concentrations (MICs) were expressed in µg/mL using an inverted mirror to display the visual growth. The MICs were expressed in µg/mL using an inverted mirror to display the visual growth. The interpretation of the results was also in accordance to recommendations provided by the CLSI [63].

### 4.4. Carbapenemases Detection by Multiplex PCR

Bacterial DNA was extracted using the boiling method, consistent with previous studies [64,65]. Subsequently, multiplex PCR was performed to assess DNA from each isolate, followed by confirmation through single PCR. Detection of clinically significant carbapenemase genes (*bla*_NDM_, *bla*_KPC_, *bla*_IMP_, *bla*_VIM_, *bla*_OXA-48_, and *bla*_GES_) was conducted using the specific primers outlined in Table 1 [66,67]. The identification of carbapenemase genes utilized GoTaq^®^ DNA polymerase (Promega, Madison, WI, USA) and a T100 thermal cycler (Bio-Rad, Hercules, CA, USA). Briefly, the multiplex PCRs were divided into two groups: group 1 included *bla*_KPC_, *bla*_VIM_, and *bla*_IMP_, while group 2 included *bla*_OXA-48_ and *bla*_NDM_. The *bla*_GES_ gene amplification was performed using a single reaction. The multiplex PCR mixture for group 1 consisted of 11.625 µL of nuclease-free water, 5 µL of 5× green GoTaq^®^ buffer, 2.5 of µL MgCL2, 0.25 µL of dNTPs, 0.5 µL of each forward primer (*bla*_KPC_, *bla*_VIM_, and *bla*_IMP_) with a concentration 10 µM, 0.5 µL of each reverse primer (*bla*_KPC_, *bla*_VIM_, and *bla*_IMP_) with a concentration of 10 µM, 0.125 µL of GoTaq^®^ DNA polymerase, and 2.5 µL of DNA template. Group 2 consisted of the same reaction mixture as before, with the only difference being the primer amounts. For this group, 0.75 µL of each forward primer (*bla*_OXA-48_ and *bla*_NDM_) and 0.75 µL of each reverse primer (*bla*_OXA-48_ and *bla*_NDM_) was used. The single PCR mixture had the same amount for each component except for the primers, which were 1.5 µL of forward primer and 1.5 µL of reverse primer. The amplification conditions for both multiplex and single PCR were the same, as follows: an initial denaturation at 95 °C for 2 min, followed by 34 cycles of amplification. Each cycle consisted of denaturation at 95 °C for 1 min, annealing at 54 °C for 40 s, and extension at 72 °C for 50 s. The final extension was at 72 °C for 5 min. Each PCR procedure included a positive and a negative control. The amplified products were analyzed by electrophoresis using a 1% agarose gel.

### 4.5. Whole-Genome Sequencing

First, a single colony from a fresh MH agar plate was suspended in 5 mL of LB broth containing 4 µg/mL of meropenem and grown overnight at 180 rpm and 37 °C. Genomic DNA was extracted from 1–2 mL of culture using the Puregene DNA extraction kit (Qiagen, CA, USA), following the manufacturer’s instructions. Genomic DNA was purified using Mag-Bind Total Pure NGS (Omega Bio-Tek, Inc., Norcross, GA, USA) to achieve the required degree of DNA purity. The genomic DNA was mixed with Mag-Bind beads at a ratio of 1.8 (beads) to 1 (genomic DNA), following the manufacturer’s instructions. Quantification and DNA purity ware determined by the ratio of absorbance at 260/280 nm and 260/230 nm using a BioDrop uLite Spectrophotometer (BioDrop, Cambridge, UK), and by fluorometry using an Invitrogen Qubit TM 4 Fluorometer (Thermo Fisher Scientific, Waltham, MA, USA). The DNA library preparation was conducted using the Rapid Barcoding Sequencing Kit (Oxford Nanopore Technologies, Oxford, UK), following the manufacturer’s instructions. The concentration of genomic DNA used for the library preparation ranged between 55 and 65 ng/µL. Sequencing was performed using MinION Mk1C devices and FLO-MIN106 flow cells for 72 h of sequencing and MinKNOW software version 21.05.26 (all provided by Oxford Nanopore Technologies, Oxford, UK).

### 4.6. Bioinformatics

Basecalling was performed using Guppy (v.6.4.6+ae70e8f) and the SUP model (dna_r9.4.1_450bps_sup.cfg), activating demultiplexing, trimming, and read splitting. The resulting reads were then further split using Duplex Sequencing Tools (v.0.2.9) [68] and quality-controlled with Filtlong (v.0.2.1) [69]. The effects of these steps were checked with NanoPlot (v.1.40.0) [70] before and after, resulting in a median of 37,406 reads, a median quality of Q14, and an N50 of 15 Kbp per sample. Flye (v.2.9-b1768) [71] was used to create assemblies, which were then polished with medaka (v.1.7.2) [72] and Homopolish (v.0.3.3) [73]. CheckM (v.1.1.3) [74] was used to assess their quality, discarding them if they had high contamination or low completeness. Species identification was performed with Kmerfinder (v.3.2) [75] and sourmash (v.4.6.1) [76]; mlst (Center for Genomic Epidemiology, v.2.0) [77] was used for ST identification. Genomes were annotated using bakta (v.1.7.0) [78], mobile elements were identified using MOB-suite (v.3.1.0) [79], and resistance genes were detected using RGI (v.5.2.0) and CARD (v.3.2.6) [80]. Additionally, Kleborate (v.2.2.0) [81] was used for *Klebsiella* characterization. To create the phylogenetic tree, PIRATE (v.1.0.4) [82] and ggtree (v.3.0.4) [83] were used.

### 4.7. Clinical Data

Data on clinical bacterial isolates detected in patients were obtained from the database of the University Hospital of A Coruña (CHUAC) (NW Spain), covering the period from 5 January 2017 to 11 September 2023.

## 5. Conclusions

Overall, our study emphasizes the role of WWTPs as a reservoir for carbapenemase genes, revealing a diverse range of bacterial sequence types (STs). Notably, the *bla*_KPC_ gene emerged as the predominant carbapenemase gene among the bacterial isolates. Significantly, this resistance gene was absent in clinical isolates at our hospital before June 2023, highlighting the importance of monitoring ARGs in WWTPs as an early warning system to anticipate potential community and hospital ARBs. However, conducting further research at WWTP sites globally is crucial to achieve a comprehensive understanding of the prevalence and diversity of carbapenemase genes worldwide. 

## Figures and Tables

**Table 1:** Oligonucleotides used in the present study.

Gene	Oligonucleotide Name	Sequence (5′-3′)	Product Size (bp)	References
*bla*_KPC_	KPC-Fm KPC-Rm	CGTCTAGTTCTGCTGTCTTG CTTGTCATCCTTGTTAGGCG	798 232	[66]
*bla*_OXA-48_	OXA-F OXA-R	GCGTGGTTAAGGATGAACAC CATCAAGTTCAACCCAACCG	438	[66]
*bla*_VIM_	VIM-F VIM-R	GATGGTGTTTGGTCGCATA CGAATGCGCAGCACCAG	390	[66]
*bla*_IMP_	IMP-F IMP-R	GGAATAGAGTGGCTTAAYTCTC GGTTTAAYAAAACAACCACC	232	[66]
*bla*_NDM_	NDM-F NDM-R	GGTTTGGCGATCTGGTTTTC CGGAATGGCTCATCACGATC	621	[66]
*bla*_GES_	GES-F GES-R	CTGGCAGGGATCGCTCACTC TTC CGATCAGCCACCTCTCA	600	[67]

## Data Availability

Further information will be provided on request.

## Supplementary Materials

The following supporting information can be downloaded at: https://www.mdpi.com/article/10.3390/antibiotics13020194/s1.
